# Circular RNA circZNF532 facilitates angiogenesis and inflammation in diabetic retinopathy via regulating miR-1243/CARM1 axis

**DOI:** 10.1186/s13098-022-00787-z

**Published:** 2022-01-21

**Authors:** Ting Wang, Chaopeng Li, Min Shi, Shi Zhou, Jiajing Chen, Fang Wang

**Affiliations:** 1 Department of Ophthalmology, The Affiliated Huaian No.1 People’s Hospital, Nanjing Medical University, Huai’an, 223300 China; 2 Department of Endocrinology, The Affiliated Huaian No.1 People’s Hospital, Nanjing Medical University, Huai’an, 223300 China; 3 Department of Ophthalmology, Shanghai Tenth People’s Hospital, Tongji University, 301 Yan’an Zhong Lu, Jing’an District, Shanghai, 200071 China

**Keywords:** Diabetic retinopathy, circZNF532, miR-1243, CARM1, High glucose

## Abstract

**Background:**

Diabetic retinopathy (DR) is a serious complication of diabetes. Numerous reports have validated that circular RNAs (circRNAs) participate in DR progression. This study aimed to elucidate the role and potential mechanism of circRNA zinc finger protein 532 (circZNF532) in DR.

**Methods:**

The levels of circZNF532, miR-1243, and coactivator associated arginine methyltransferase 1 (CARM1) in DR patients and human retinal microvascular endothelial cells (hRMECs) were determined by quantitative real-time PCR and western blot. Colony formation assay, transwell assay, tube formation assay and enzyme-linked immunosorbent assay were used to assess the biological function of hRMECs. The binding relationship between miR-1243 and circZNF532/CARM1 was verified by dual-luciferase reporter and RNA immunoprecipitation assays.

**Results:**

circZNF532 and CARM1 levels were increased, while miR-1243 level was reduced in DR patients and high glucose (HG)-stimulated hRMECs. In terms of mechanism, miR-1243 competitively bound to circZNF532 and CARM1. Down-regulation of circZNF532 restrained HG-induced hRMECs proliferation, migration, invasion, angiogenesis and inflammation via regulating miR-1243. In addition, miR-1243 inhibited HG-triggered hRMECs progression via targeting CARM1.

**Conclusion:**

circZNF532 facilitated HG-induced angiogenesis and inflammation in hRMECs via modulating the miR-1243/CARM1 pathway, suggesting that circZNF532 might be a potential biomarker for DR treatment.

**Supplementary Information:**

The online version contains supplementary material available at 10.1186/s13098-022-00787-z.

## Introduction

Diabetic retinopathy (DR) is a common microvascular complication of diabetes leading to impaired vision and blindness, and one-third of diabetic patients suffer from DR [1]. It is estimated that the population of patients with diabetic eye disease in Europe will increase to 8.6 million in 2050 [2]. Hyperglycemia induces retinal endothelial dysfunction, which in turn promotes the progression of DR [3, 4]. The hallmarks of DR are angiogenesis, oxidative stress, and inflammation [5, 6]. In recent years, the incidence of DR has been increasing. Although the treatment of DR has made great progress, DR is still an important cause of vision loss worldwide [7]. Hence, elucidating the pathological mechanism of DR is essential for developing new clinical treatment strategies for DR.

Circular RNAs (circRNAs) are novel endogenous non-coding RNAs with circular structures formed by back-splicing [8]. Existing studies have identified that circRNAs participate in regulating diverse biological events, including invasion, migration, vascularization, and inflammation [9, 10]. In addition, the aberrant expression of circRNAs is related to the pathogenesis of various diseases, like cancers, immune diseases, and metabolic diseases [11–13]. Mounting reports have validated that circRNAs exert their functional effects in different diseases via functioning as microRNA (miRNA) sponges [14]. Additionally, miRNAs play crucial roles in multiple diseases through combining with mRNAs to repress their translation and stability [15]. Besides, several reports have corroborated that circRNAs are identified as regulators of DR progression [16]. For instance, circRNA_0084043 facilitated high glucose-resulted damage in ARPE-19 cells by promoting inflammation and oxidative stress [17]. Zou et al. revealed that circCOL1A2 contributed to angiogenesis in DR via elevating VEGF expression through combining with miR-29b [18]. A previous study found that circRNA zinc finger protein 532 (circZNF532; hsa_circ_0047814) was strikingly up-regulated in DR through circular RNA microarrays [19]. Nonetheless, the potential effects and mechanism of circZNF532 in DR are still largely unknown. Bioinformatics prediction showed that circZNF532 may interact with miR-1243, which is prominently decreased in DR patients [20]. Nevertheless, the relationship between circZNF532 and miR-1243 in DR progression remains indistinct.

In this research, we were committed to detecting the expression pattern of circZNF532 in DR patients and high glucose-triggered hRMECs. Furthermore, we explored the biological function and potential mechanism of circZNF532 in high glucose-disposed hRMECs, suggesting that the circZNF532/miR-1243/coactivator associated arginine methyltransferase 1 (CARM1) axis might provide new biomarkers for DR treatment.

## Materials and methods

### Clinical samples

DR patients (n = 23) and healthy volunteers (n = 23) were recruited from The Affiliated Huaian No.1 People’s Hospital, Nanjing Medical University. Serum samples were collected from all DR patients and healthy controls. According to the DR staging criteria, DR patients were divided into stage IV, stage V and stage VI. All participants were informed and signed written informed consent. This research was ratified by the Ethics Committee of The Affiliated Huaian No.1 People’s Hospital, Nanjing Medical University. The clinicopathological parameters of DR and control groups are presented in Table 1.

**Table 1:** Comparison of clinical data between DR and Control groups

Characteristics	Control (n = 23)	DR(n = 23)	P value
Gender (Male/female)	15/8	16/7	0.55
Age (years)	51.52 ± 6.2	50.38 ± 5.8	0.62
BMI (kg/m^2^)	28.3 ± 3.6	25.9 ± 5.2	0.01^*^
Smoking history			0.09
No	18	16	
Yes	5	7	
HbA1c (%)	3.86 ± 0.41	8.96 ± 2.33	0.04^*^
Family history of DM			0.42
No	15	16	
Yes	8	7	
BP systolic, mm Hg	103.5 ± 6.98	118.4 ± 14.21	0.03^*^
BP diastolic, mm Hg	63.2 ± 8.99	75.23 ± 10.15	0.02^*^
TG (mmol/L)	1.38 ± 0.21	1.53 ± 0.18	0.63
TC (mmol/L)	4.02 ± 0.47	4.36 ± 0.48	0.06
HDL (mg/dL)	58.31 ± 11.96	50.12 ± 12.31	0.03^*^
LDL (mg/dL)	126.25 ± 21.21	136.33 ± 12.89	0.06
Urinary creatinine, mmol/L	9.62 ± 5.11	11.32 ± 4.52	0.46
FPG (mmol/L)	5.03 ± 0.85	7.62 ± 0.38	0.02^*^
FINS (mIU/I)	5.36 ± 0.68	5.52 ± 0.77	0.51

BMI, body mass index; HbA1c, haemoglobin A1c; DM, diabetes mellitus; DR, diabetic retinopathy; BP, blood pressure; TG, triacylglycerol; TC, total cholesterol; HDL, high-density lipoprotein; LDL, low density lipoprotein; FPG, fasting plasma glucose; FINS, fasting insulin

^*^P < 0.05

### Cell culture

Human RMECs (hRMECs) were purchased from Yuchicell Biological Technology Co., Ltd. (Shanghai, China) and cultured in endothelial cell medium (ECM; ScienCell, San Diego, CA, USA) containing 10% fetal bovine serum (FBS; Hyclone, Logan, UT, USA) with 5% CO_2_ at 37 °C. To simulate a high glucose environment, hRMECs were exposed to 25 mM glucose (HG; Solarbio, Beijing, China). Additionally, hRMECs were stimulated with 5.5 mM glucose (NG; Solarbio) as the control group.

### Cell transfection

circZNF532 small interfering RNA (si-circZNF532) and the control (si-NC), miR-1243 mimics (miR-1243) and the control (miR-NC), miR-1243 inhibitor (in-miR-1243) and the control (in-miR-NC), CARM1 overexpression vector (CARM1) and the control (pcDNA) were purchased from GenePharma (Shanghai, China). Lipofectamine 3000 (Invitrogen, Carlsbad, CA, USA) was employed for cell transfection when hRMECs reached ~ 80% confluence.

### Quantitative real-time PCR (qRT-PCR)

Total RNA was obtained using TRIzol reagent (Leagene, Beijing, China). Then, RNase R (3 U/μg; Seebio, Shanghai, China) treatment was implemented to detect the circular characteristics of circZNF532. Subsequently, cDNA was synthesized using the specific cDNA synthesis kit (Vazyme, Nanjing, China). Next, qRT-PCR reactions were performed using SYBR Green Master Mix (Vazyme). 2^−ΔΔCt^ method was utilized to calculate gene expression. The primers included: circZNF532-F: 5′-ACGAGTGGACAAAACATCTGC-3′, circZNF532-R: 5′-AATGCTGCCAGGAGGTCATC-3′; ZNF532-F: 5′-ACTGGCAATGGCTTACATAATGG-3′, ZNF532-R: 5′-CTGGCTGAATGTCGAGTCTTT-3′; miR-1243-F: 5′-AACTGGATCAATTATA-3′, miR-1243-R: 5′-GTGCAGGGTCCGAGGT-3′; CARM1-F: 5′-TCGCCACACCCAACGATTT-3′, CARM1-R: 5′-GTACTGCACGGCAGAAGACT-3′; GAPDH-F: 5′-GCACCGTCAAGGCTGAGAAC-3′, GAPDH-R: 5′-ATGGTGGTGAAGACGCCAGT-3′; U6-F: 5′-CTCGCTTCGGCAGCACATA-3′, U6-R: 5′-AACGCTTCACGAATTTGCGT-3′. GAPDH and U6 were used as endogenous controls.

### Colony formation assay

After digestion with 0.25% trypsin (Solarbio), hRMECs were plated into 6-well plates. After incubation for 12 days, hRMECs were fixed with 4% paraformaldehyde and stained with 0.1% crystal violet (Solarbio). Afterwards, the colonies were counted under a microscope (Olympus, Tokyo, Japan) at 100× magnification.

### Transwell assay

Cell migration and invasion capabilities were determined using 24-well transwell chambers with or without Matrigel (Corning Life Sciences, Corning, NY, USA). Briefly, hRMECs were cultured in the upper chamber, and the bottom chamber was filled with 500 µL of medium containing 10% FBS (Hyclone). Following 24 h of culture, the cells on the lower surface were fixed with 4% paraformaldehyde (Solarbio) and stained with 0.5% crystal violet (Solarbio). Afterwards, the cells were observed and counted under a microscope (Olympus) at 100× magnification.

### Tube formation assay

The treated hRMECs were inoculated into 24-well plates pre-coated with growth factor reduced (GFR) Matrigel Matrix (Corning Life Sciences). Following 16 h of culture, the capillary-like structures were photographed in five random fields under a microscope (Olympus) and measured using ImageJ software (National Institutes of Health, Bethesda, MD, USA).

### Enzyme-linked immunosorbent assay (ELISA)

Following treatment, hRMECs medium was collected. Subsequently, the concentrations of interleukin 1 beta (IL-1β), IL-6, and tumor necrosis factor alpha (TNF-α) were measured using corresponding ELISA kits (R&D Systems, Minneapolis, MN, USA) according to the manufacturer’s instructions.

### Dual-luciferase reporter assay

The sequences of circZNF532 or CARM1 3′UTR containing wild-type or mutant miR-1243 binding sites were inserted into pmirGLO vector (Promega, Madison, WI, USA) to form circZNF532 WT, circZNF532 MUT, CARM1 3′UTR WT or CARM1 3′UTR MUT. Subsequently, the constructed vector and miR-1243 or miR-NC were co-transfected into hRMECs. The luciferase activity was examined via Dual-Lucy Assay Kit (Solarbio).

### RNA immunoprecipitation (RIP) assay

RIP analysis was implemented using EZ-Magna RIP kit (Millipore, Billerica, MA, USA). After lysing cells with RIP lysis buffer, cell lysates were cultured with magnetic beads combined with anti-Ago2 or anti-IgG (negative control). The levels of circZNF532 and miR-1243 in the precipitate were determined using qRT-PCR.

### Western blot assay

Following extracting with RIPA lysis buffer (Solarbio), the protein was quantified using BCA Protein Assay Kit (Beyotime, Shanghai, China). Afterwards, the equal amount of protein was separated by 10% SDS-PAGE and transferred to PVDF membranes (Millipore). After blocking for 2 h in 5% skimmed milk, the membranes were probed with primary antibodies against CARM1 (1:1500, ab128851, Abcam) or β-actin (1:2000, ab8227, Abcam). Subsequently, the membranes were probed with HRP-conjugated secondary antibody (1:25,000, ab205718, Abcam). At last, the protein bands were detected using ECL reagent (Absin, Shanghai, China).

### Statistical analysis

Data were expressed as mean ± standard deviation in three independent replicates by using GraphPad Prism 7 software (GraphPad, San Diego, CA, USA). The differences were analyzed using Student’s *t*-test or one-way analysis of variance. *P* < 0.05 was considered statistically significant.

## Results

### Expression of circZNF532 in DR patients

**Fig. 1:**
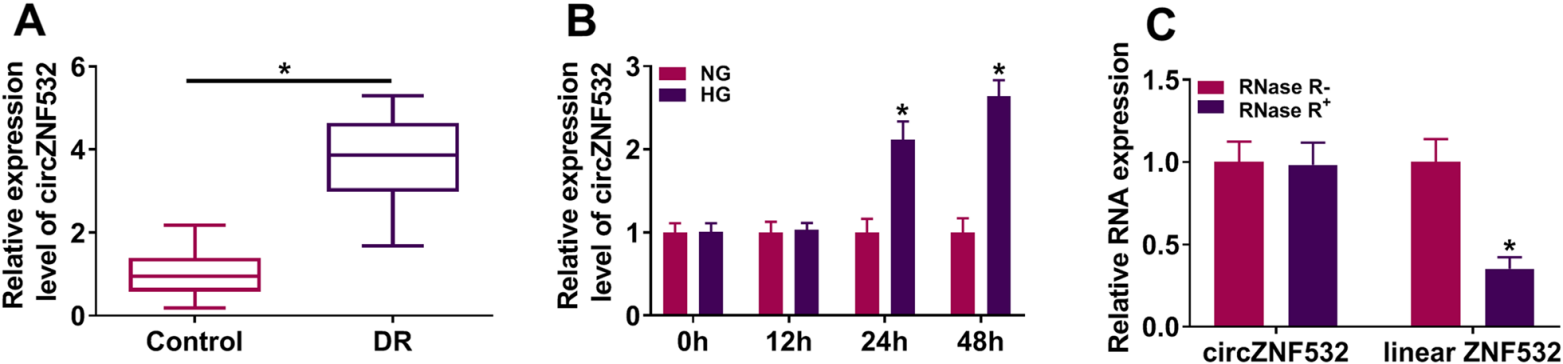
Expression of circZNF532 in DR patients and high glucose-treated hRMECs. **A** The expression of circZNF532 in serum from DR patients (n = 23) and healthy controls (n = 23) was detected using qRT-PCR. **B** hRMECs were exposed to 5.5 mM glucose (normal glucose, NG) or 25 mM glucose (high glucose, HG) for different times, and circZNF532 level was measured using qRT-PCR. **C** The levels of circZNF532 and linear ZNF532 were examined by qRT-PCR after RNase R treatment. *P < 0.05

First of all, we confirmed the expression of circZNF532 in serum samples from DR patients. As depicted in Fig. 1A and Additional file 2: Fig. S2A, circZNF532 level was prominently increased in serum samples from DR patients compared to the control groups. As shown in Additional file 1: Fig. S1A, circZNF532 level was the highest in serum samples from DR patients at stage VI. These results showed that circZNF532 might be related to DR.

### CircZNF532 was overexpressed in HG-treated hRMECs

Subsequently, the effect of glucose on circZNF532 expression in vitro was investigated. The results exhibited that glucose treatment increased circZNF532 expression in hRMECs in a dose-dependent manner (Additional file 1: Fig. S1B). Moreover, the level of circZNF532 was examined after hRMECs were exposed to 5.5 mM glucose (NG) or 25 mM glucose (HG) for different times (0, 12, 24, and 48 h). Compared with the NG group, circZNF532 level in the HG group was remarkably increased after 24 h and 48 h treatment (Fig. 1B). Besides, RNase R digestion was performed in hRMECs to detect the circular feature of circZNF532. As illustrated in Fig. 1C, circZNF532 was more resistant to RNase R than linear ZNF532, suggesting that circZNF532 was a stable circular RNA. Furthermore, hemoglobin A1c (HbA1c), blood pressure (BP) systolic, BP diastolic, and fasting plasma glucose (FPG) were significantly higher in DR patients than that in the control group, while body mass index (BMI) and high-density lipoprotein (HDL) were markedly lower in DR patients than that in control group (Table 1). These data indicated that circZNF532 might be involved in the dysfunction of hRMECs under HG environment.

### Depletion of circZNF532 alleviated proliferation, migration, invasion, tube formation and pro-inflammatory cytokine release induced by HG in hRMECs

**Fig. 2:**
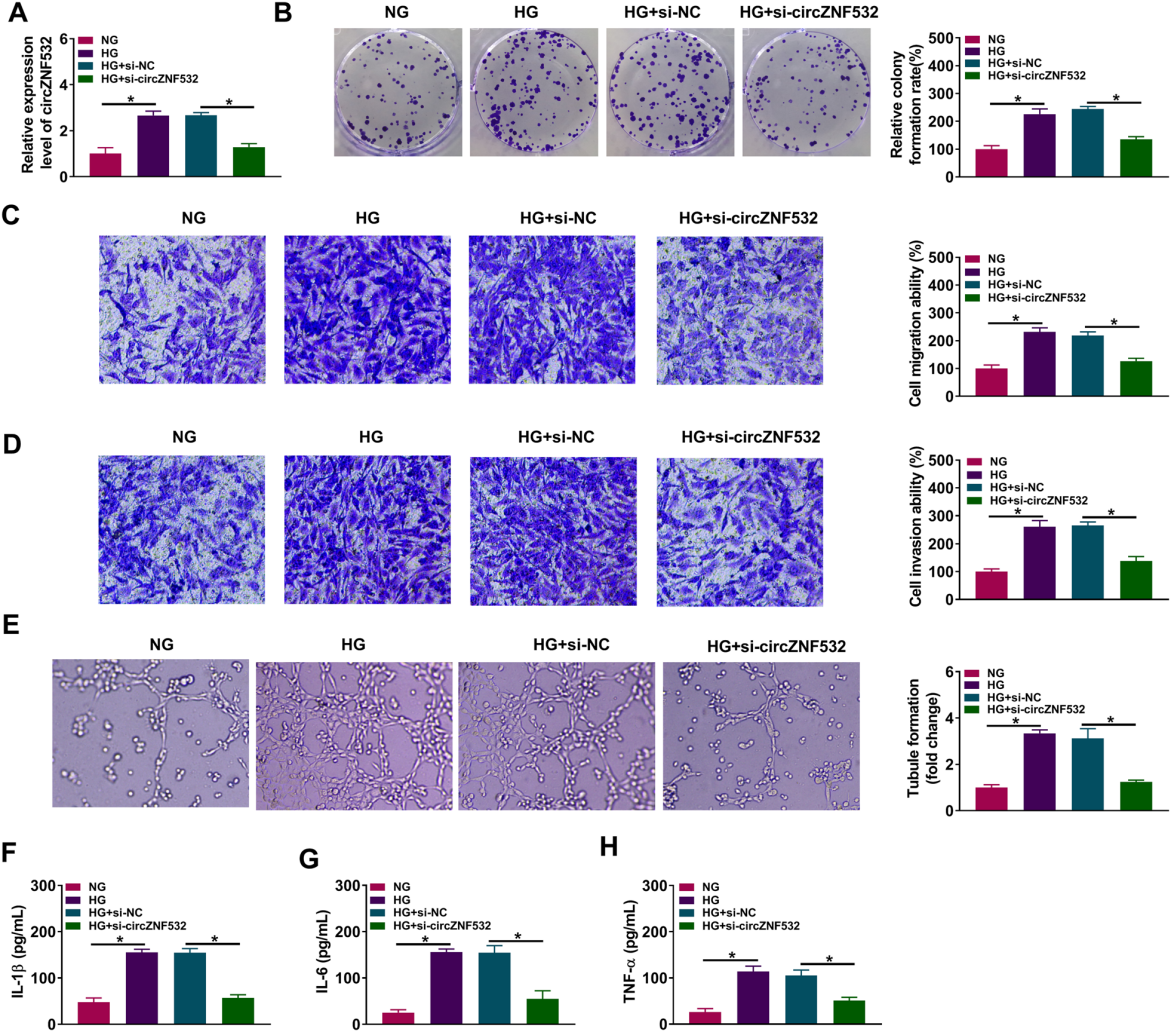
Depletion of circZNF532 alleviated proliferation, migration, invasion, tube formation and pro-inflammatory cytokine release induced by HG in hRMECs. After si-circZNF532 or si-NC transfection, hRMECs were treated with NG or HG for 48 h. **A** The expression of circZNF532 was examined by qRT-PCR. **B** Cell proliferation was tested by colony formation assay. **C** and **D** Cell migration and invasion were assessed by transwell assay. **E** Tube formation assay was utilized to evaluate angiogenesis. **F**–**H** The levels of pro-inflammatory cytokines (IL-1β, IL-6 and TNF-α) were measured using ELISA assay. *P < 0.05

To evaluate the role of circZNF532 in hRMECs under HG stimulation, hRMECs were exposed to NG or HG for 48 h following si-circZNF532 or si-NC transfection. First, qRT-PCR analysis showed that introduction of si-circZNF532 significantly reduced the increase in circZNF532 level induced by HG treatment (Fig. 2A). Colony formation assay revealed that HG stimulation markedly elevated the proliferation ability of hRMECs compared to the NG group, while this impact was abolished by inhibiting circZNF532 (Fig. 2B). Transwell analysis showed that HG exposure strikingly increased the migration and invasion capabilities of hRMECs compared with the NG group, whereas down-regulation of circZNF532 reversed this impact (Fig. 2C and D). In addition, HG treatment promoted the tube formation of hRMECs in comparison with NG stimulation, which was abrogated after introduction of si-circZNF532 (Fig. 2E). Furthermore, ELISA assay indicated that the levels of pro-inflammatory cytokines (IL-1β, IL-6 and TNF-α) were markedly increased in the HG group compared with the NG group, while circZNF532 silencing restrained the release of pro-inflammatory cytokines (Fig. 2F–H). These results suggested that interference of circZNF532 suppressed proliferation, migration, invasion, angiogenesis and inflammation induced by HG stimulation in hRMECs.

### circZNF532 served as a molecular sponge for miR-1243

**Fig. 3:**
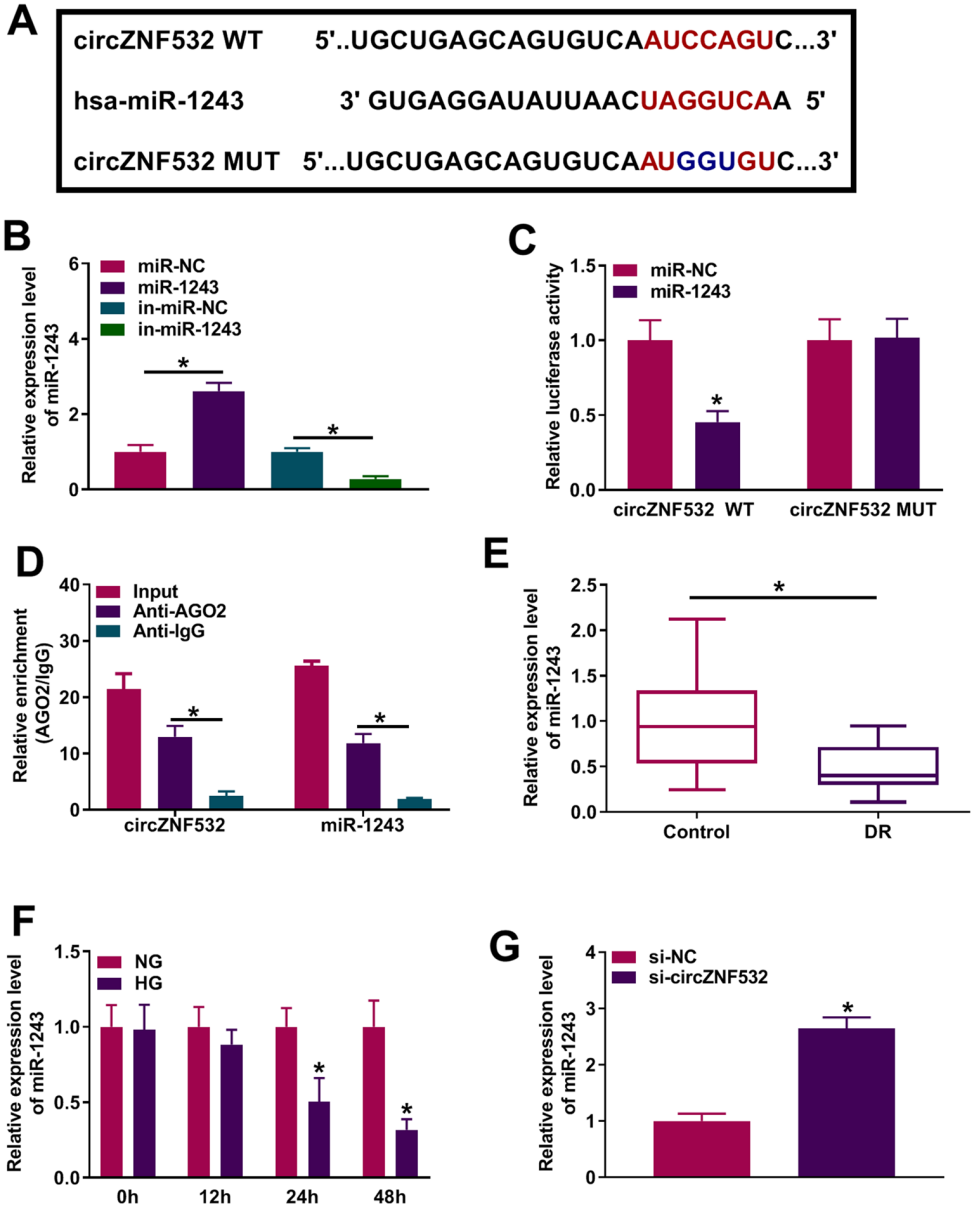
circZNF532 served as a molecular sponge for miR-1243. **A** The predicted binding site between circZNF532 and miR-1243 was displayed by circinteractome. **B** The overexpression and knockdown efficiencies of miR-1243 were determined using qRT-PCR. **C** and **D** The binding relationship between circZNF532 and miR-1243 was validated by dual-luciferase reporter and RIP assays. **E** The level of miR-1243 in serum from DR patients (n = 23) and healthy controls (n = 23) was examined by qRT-PCR. **F** The expression of miR-1243 was detected by qRT-PCR in hRMECs stimulated with NG or HG for different times. **G** The level of miR-1243 was measured using qRT-PCR in hRMECs transfected with si-circZNF532 or si-NC. *P < 0.05

Bioinformatics software (https://circinteractome.irp.nia.nih.gov/) predicted the possible binding sequence between circZNF532 and miR-1243 (Fig. 3A). Subsequently, qRT-PCR analysis showed that miR-1243 overexpression and knockdown efficiencies were both significant (Fig. 3B). Dual-luciferase reporter assay revealed that co-transfection of miR-1243 and circZNF532 WT remarkably reduced the luciferase activity of hRMECs (Fig. 3C). RIP analysis showed that circZNF532 and miR-1243 were significantly enriched in the anti-AGO2 group compared to the anti-lgG group (Fig. 3D). In addition, miR-1243 level in DR patients was markedly reduced compared with healthy controls (Fig. 3E and Additional file 2: Fig. S2B). Also, HG remarkably decreased miR-1243 expression in a time-dependent manner, while NG had no significant effect on miR-1243 expression (Fig. 3F). As displayed in Additional file 1: Fig. S1C, miR-1243 level was significantly decreased in a dose-dependent manner. Besides, circZNF532 down-regulation strikingly elevated the expression of miR-1243 (Fig. 3G). These data demonstrated that circZNF532 directly sponged miR-1243.

### circZNF532 modulated the function of hRMECs via sponging miR-1243

To investigate the role of circZNF532/miR-1243 axis in hRMECs stimulated by HG, hRMECs were introduced with si-circZNF532 or/and in-miR-1243 and then exposed to 25 mM glucose for 48 h. As presented in Additional file 3: Fig. S3A, co-transfection of in-miR-1243 and si-circZNF532 alleviated the increase in miR-1243 level triggered by circZNF532 knockdown alone in HG-disposed hRMECs. In addition, depletion of circZNF532 hindered cell proliferation (Additional file 3: Fig. S3B), migration (Additional file 3: Fig. S3C), invasion (Additional file 3: Fig. S3D) and tube formation (Additional file 3: Fig. S3E) in HG-exposed hRMECs, while these changes were restored by down-regulating miR-1243. Moreover, circZNF532 silence significantly reduced the release of pro-inflammatory cytokines in HG-stimulated hRMECs, which was overturned after transfection with in-miR-1243 (Additional file 3: Fig. S3F–H). These data evidenced that circZNF532 silencing weakened the dysfunction of hRMECs by modulating miR-1243 under HG stimulation.

### circZNF532 indirectly regulated CARM1 by binding to miR-1243

**Fig. 4:**
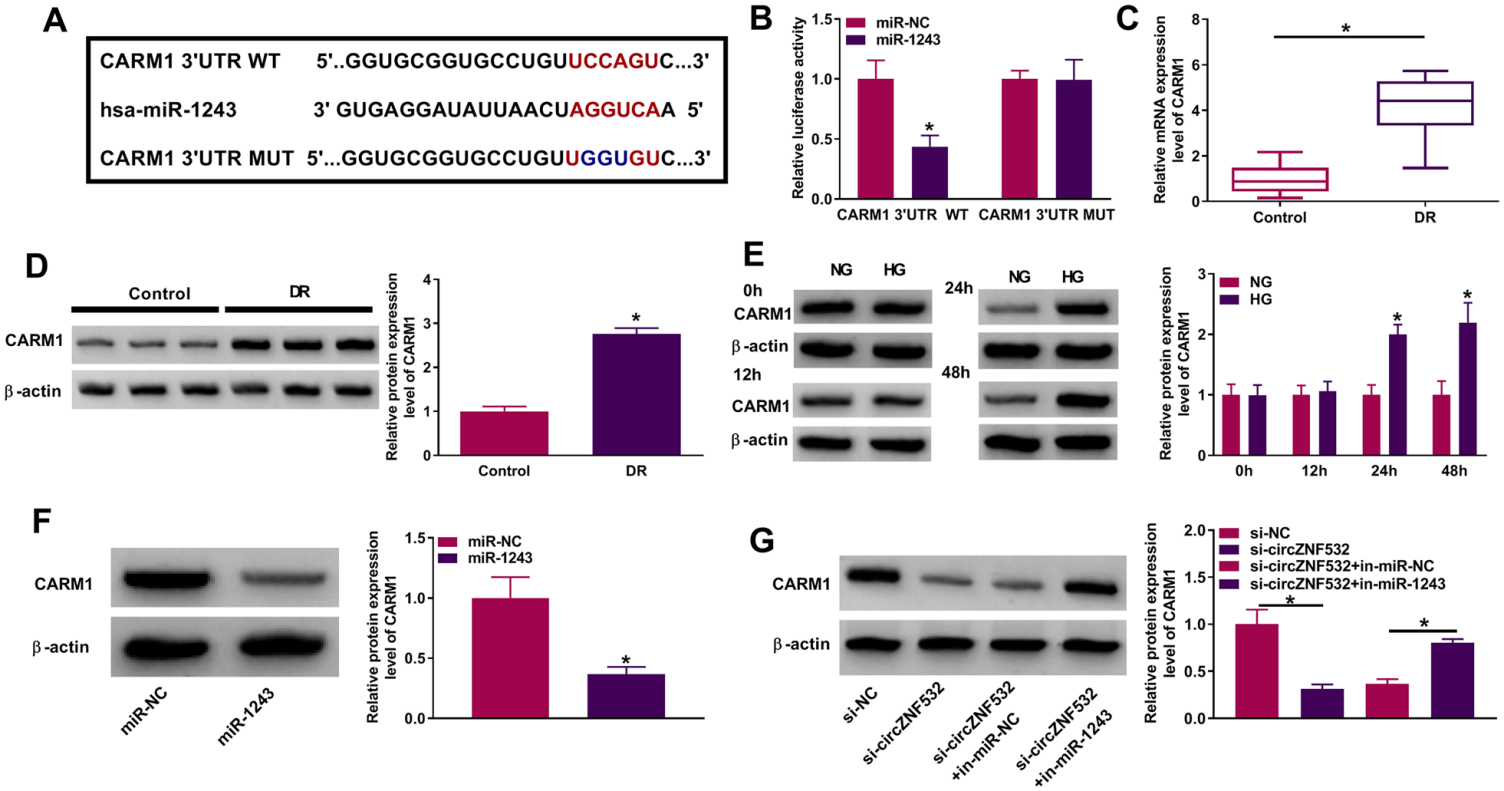
circZNF532 indirectly regulated CARM1 by binding to miR-1243. **A** The putative binding site of miR-1243 on CARM1 3′UTR was exhibited. **B** The interaction between miR-1243 and CARM1 was determined by dual-luciferase reporter assay. **C** and **D** CARM1 mRNA and protein levels in serum from DR patients (n = 23) and healthy controls (n = 23) were examined by qRT-PCR and western blot. **E** CARM1 protein expression was measured by western blot in hRMECs treated with NG or HG for different times. **F** CARM1 protein level was examined by western blot in hRMECs transfected with miR-NC or miR-1243. **G** hRMECs were introduced with si-NC, si-circZNF532, si-circZNF532 + in-miR-NC or si-circZNF532 + in-miR-1243, and CARM1 protein level was detected using western blot. *P < 0.05

Next, the microT-CDS online database (http://diana.imis.athena-innovation.gr/DianaTools/index.php?r=microT_CDS/index) predicted that miR-1243 and CARM1 3′UTR possessed putative binding sites (Fig. 4A). Dual-luciferase reporter assay revealed that miR-1243 mimics strikingly decreased the luciferase activity of CARM1 3′UTR WT (Fig. 4B). Compared with healthy controls, CARM1 mRNA and protein levels were markedly increased in DR patients (Fig. 4C, D and Additional file 2: Fig. S2C). In the meantime, HG treatment conspicuously elevated CARM1 protein expression in a time-dependent manner relative to NG exposure (Fig. 4E). Furthermore, CARM1 level was remarkably elevated in a dose-dependent manner (Additional file 1: Fig. S1D). Additionally, introduction of miR-1243 mimics markedly reduced CARM1 protein level compared with the miR-NC group (Fig. 4F). Moreover, co-transfection of si-circZNF532 and in-miR-1243 restored the decrease in CARM1 protein level caused by circZNF532 knockdown alone (Fig. 4G). These data indicated that circZNF532 indirectly up-regulated CARM1 via absorbing miR-1243.

### CARM1 reversed the repressive effect of miR-1243 on HG-induced hRMEC function

To elucidate the effect of miR-1243/CARM1 axis on DR progression, hRMECs were stimulated with 25 mM glucose after transfection with miR-1243 or/and CARM1. First of all, western blot assay suggested that introduction of miR-1243 prominently reduced the protein expression of CARM1, while this change was restored through co-transfection with miR-1243 and CARM1 (Additional file 4: Fig. S4A). Besides, augmentation of miR-1243 suppressed cell proliferation (Additional file 4: Fig. S4B), migration (Additional file 4: Fig. S4C), invasion (Additional file 4: Fig. S4D) and tube formation (Additional file 4: Fig. S4E) in HG-treated hRMECs, whereas these impacts were mitigated via up-regulating CARM1. Furthermore, miR-1243 overexpression remarkably restrained the release of inflammatory cytokines in HG-disposed hRMECs, while up-regulation of CARM1 abrogated this impact (Additional file 4: Fig. S4F–H). Collectively, these data demonstrated that miR-1243 hindered HG-induced hRMEC dysfunction via targeting CARM1.

## Discussion

Hyperglycemia has been identified as one of the metabolic factors of life-threatening microvascular and macrovascular complications in diabetic patients [21]. Persistent hyperglycemia causes changes in microvascular morphology, which in turn leads to endothelial cell proliferation and neovascularization, which leads to DR [22]. In the present research, we clarified the role of circZNF532 in DR progression. As expected, circZNF532 regulated HG-mediated microvascular endothelial cell dysfunction in hRMECs. In terms of mechanism, we revealed a new competitive endogenous RNA (ceRNA) network involving circZNF532.

Numerous investigations have verified that circRNAs reverse the repressive effect of miRNAs on their target genes via serving as miRNA sponges [10]. Also, dysregulated circRNAs in DR patients can affect the development of DR through the ceRNA mechanism. For example, hsa_circ_0041795 aggravated human retinal pigment epithelial cell damage caused by HG by competitively binding to miR-646 and up-regulating VEGFC [23]. Zhu et al. showed that depletion of circDNMT3B triggered diabetic retinal vascular dysfunction via modulating miR-20b-5p/BAMBI pathway [24]. In addition, Jiang et al. suggested that circZNF532 was conspicuously up-regulated in the vitreous humor of diabetic patients, and its high expression could attenuate vascular dysfunction induced by diabetes through repressing miR-29a-3p [25]. In this research, we elucidated that circZNF532 expression was prominently increased in DR patients and HG-treated hRMECs. Furthermore, down-regulation of circZNF532 suppressed HG-resulted proliferation, migration, invasion, angiogenesis and inflammation in hRMECs.

Moreover, we further studied the potential mechanism of circZNF532 in DR progression, and selected miR-1243 as a possible target of circZNF532 based on previous reports and bioinformatics analysis. MiR-1243 could weaken HG-triggered hRMEC proliferation, migration, and angiogenesis, and circ_0002570 sponged miR-1243 to elevate angiomotin expression, thus contributing to hRMEC proliferation, migration, and angiogenesis under HG treatment [20]. Consistently, we also demonstrated that miR-1243 could weaken hRMEC proliferation, migration, and angiogenesis under HG stimulation. In addition, we also showed that miR-1243 decreased HG-induced inflammatory response in hRMECs, and miR-1243 depletion overturned the impact of circZNF532 knockdown on retinal endothelial dysfunction, suggesting that circZNF532 mediated retinal endothelial dysfunction through interacting with miR-1243. At present, there are very few studies on miR-1243 involved in HG, diabetes, and diabetes-related diseases, and further investigation is needed.

Furthermore, our research first demonstrated that miR-1243 could target CARM1. CARM1 is a transcriptional co-activator belonging to the protein arginine methyltransferase family [26, 27]. CARM1 accelerated retinal pigment epithelial cell apoptosis by mediating H3R17 asymmetric dimethylation, thereby promoting the progression of DR [28]. Additionally, Guo et al. revealed that CARM1 up-regulation expedited cell apoptosis in HG-stimulated retinal pigment epithelial cells via binding to miR-542-5p [29]. In our research, we verified that CARM1 level was overtly elevated in DR patients and HG-disposed hRMECs. More importantly, miR-1243 impeded the proliferation, migration, invasion, angiogenesis and inflammation of hRMECs through negatively regulating CARM1 under HG stimulation. Regrettably, the signal pathways related to this study have not been explored, and this needs to be further explored in the future.

## Conclusions

**Fig. 5:**
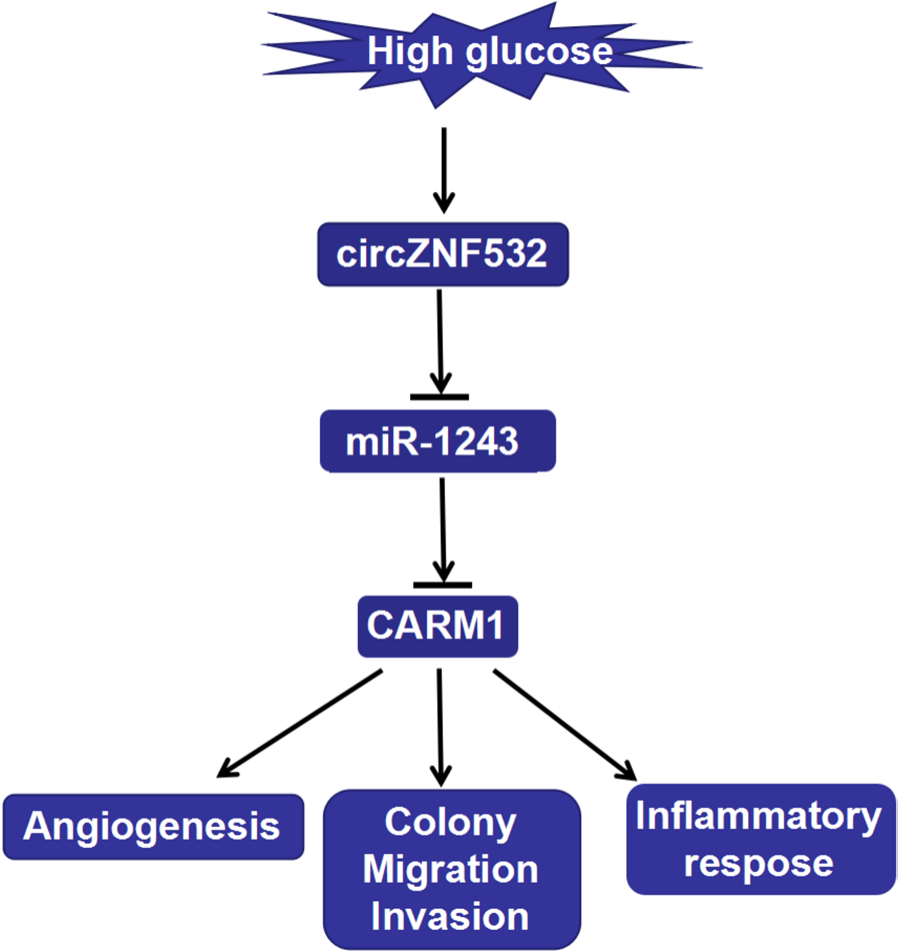
Molecular mechanism of circZNF532/miR-1243/CARM1 axis in DR. The flow chart displayed the mechanism by which circZNF532 regulated DR progression through miR-1243/CARM1 axis

In conclusion, our research corroborated that circZNF532 expedited HG-indcued hRMEC dysfunction  via sponging miR-1243 and activating CARM1 (Fig. 5). These findings might provide promising therapeutic targets for DR therapy. The limitation of this work is the lack of in vivo experiments to verify the role of circZNF532 in DR.

## Supplementary Information

**Additional file 1: Figure S1.** (A) The expression level of circZNF532 in vitreous tissues of DR patients at different stages was detected using qRT-PCR. (B-D) hRMECs were exposed to different concentrations of glucose (5.5, 10, 15, 20 and 25 mM), and the levels of circZNF532, miR-1243 and CARM1 were examined by qRT-PCR. *P < 0.05.

**Additional file 2: Figure S2.** The levels of circZNF532, miR-1243 and CARM1 in serum from DR patients (n = 23) and healthy controls (n = 23) were measured by qRT-PCR.

**Additional file 3: Figure S3.** circZNF532 modulated the function of hRMECs via sponging miR-1243. hRMECs were introduced with si-circZNF532 or/and in-miR-1243, and then stimulated with high glucose for 48 h. (A) The expression of miR-1243 was detected by qRT-PCR. Cell proliferation (B), migration and invasion (C and D), angiogenesis (E) and pro-inflammatory cytokine release (F–H) were assessed by colony formation, transwell, tube formation and ELISA assays, respectively. *P < 0.05.

**Additional file 4: Figure S4.** CARM1 reversed the repressive effect of miR-1243 on high glucose-induced hRMECs function. Following transfection with miR-1243 or/and CARM1, hRMECs were exposed to high glucose for 48 h. Western blot, colony formation, transwell, tube formation and ELISA assays were applied to detect CARM1 protein level (A), cell proliferation (B), migration and invasion (C and D), angiogenesis (E) and pro-inflammatory cytokine release (F–H), respectively. *P < 0.05.

## Data Availability

Not applicable.
