# Supplementary figures and images for: Circular RNA circZNF532 facilitates angiogenesis and inflammation in diabetic retinopathy via regulating miR-1243/CARM1 axis
Source: Diabetol Metab Syndr. 2022 Jan 21;14:14. doi: 10.1186/s13098-022-00787-z (PMC8780307; doi:10.1186/s13098-022-00787-z)

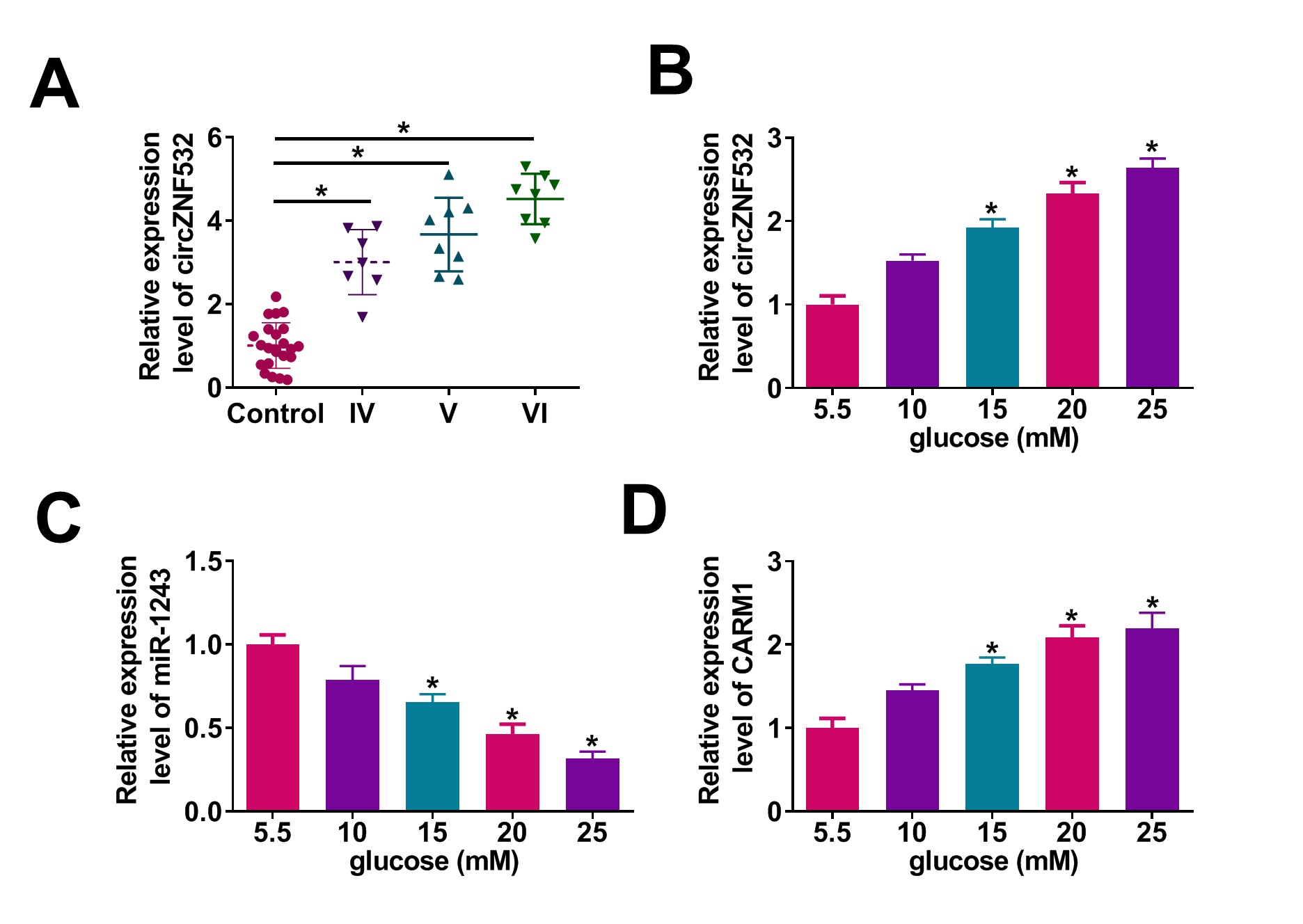

Supplement: Supplementary file 1 — Additional file 1: Figure S1. (A) The expression level of circZNF532 in vitreous tissues of DR patients at different stages was detected using qRT-PCR. (B-D) hRMECs were exposed to different concentrations of glucose (5.5, 10, 15, 20 and 25 mM), and the levels of circZNF532, miR-1243 and CARM1 were examined by qRT-PCR. *P < 0.05. [file 13098_2022_787_MOESM1_ESM.tif]

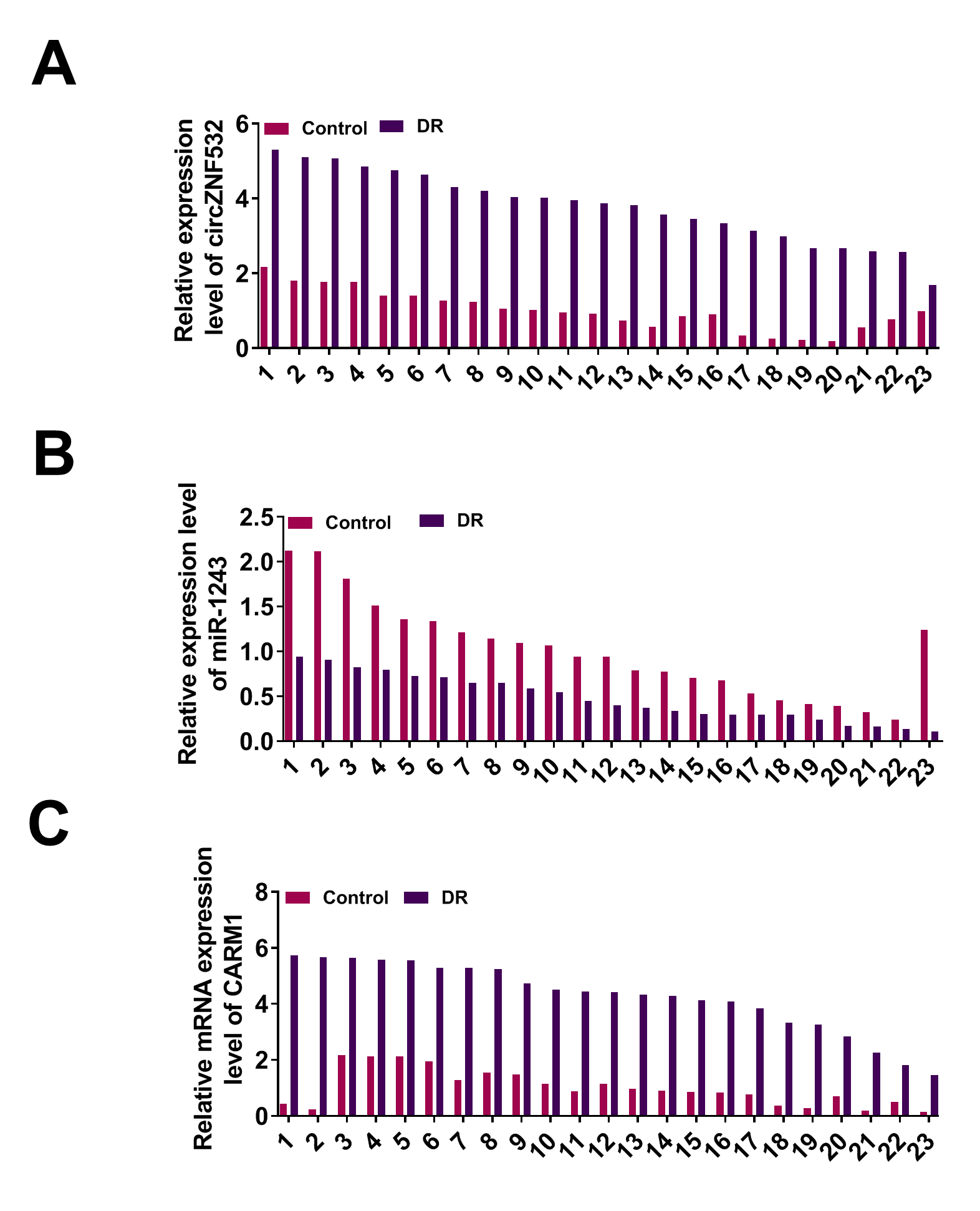

Supplement: Supplementary file 2 — Additional file 2: Figure S2. The levels of circZNF532, miR-1243 and CARM1 in serum from DR patients (n = 23) and healthy controls (n = 23) were measured by qRT-PCR. [file 13098_2022_787_MOESM2_ESM.tif]

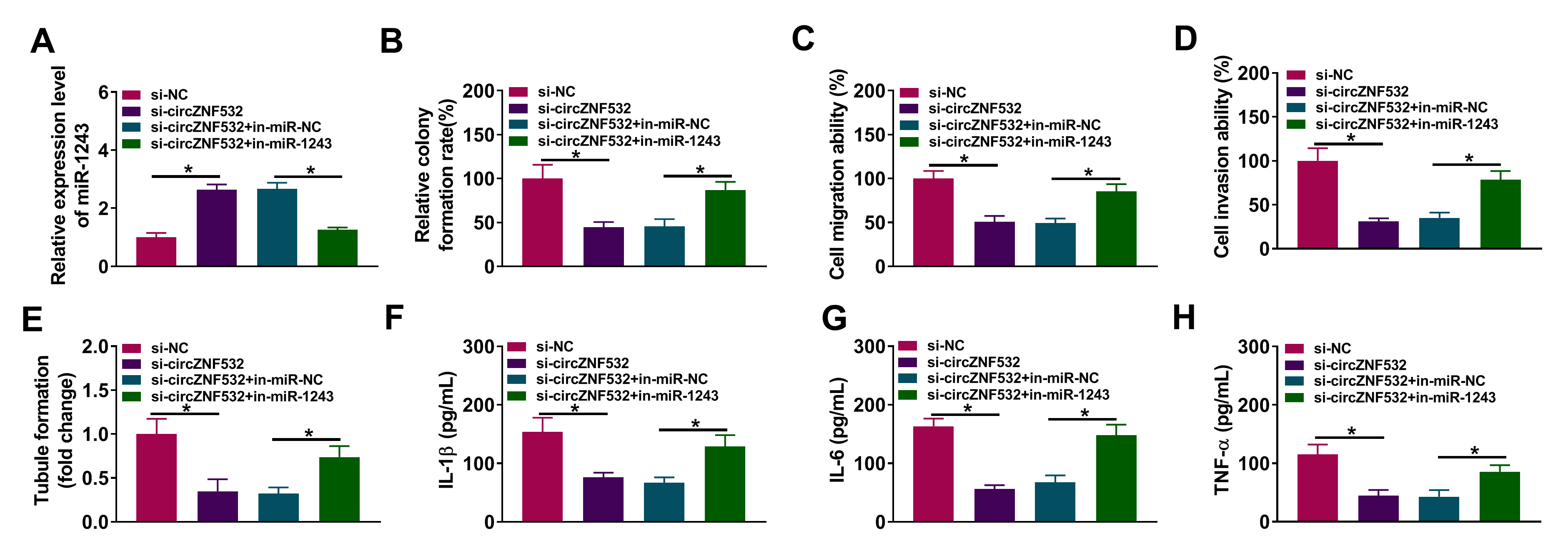

Supplement: Supplementary file 3 — Additional file 3: Figure S3. circZNF532 modulated the function of hRMECs via sponging miR-1243. hRMECs were introduced with si-circZNF532 or/and in-miR-1243, and then stimulated with high glucose for 48 h. (A) The expression of miR-1243 was detected by qRT-PCR. Cell proliferation (B), migration and invasion (C and D), angiogenesis (E) and pro-inflammatory cytokine release (F–H) were assessed by colony formation, transwell, tube formation and ELISA assays, respectively. *P < 0.05. [file 13098_2022_787_MOESM3_ESM.tif]

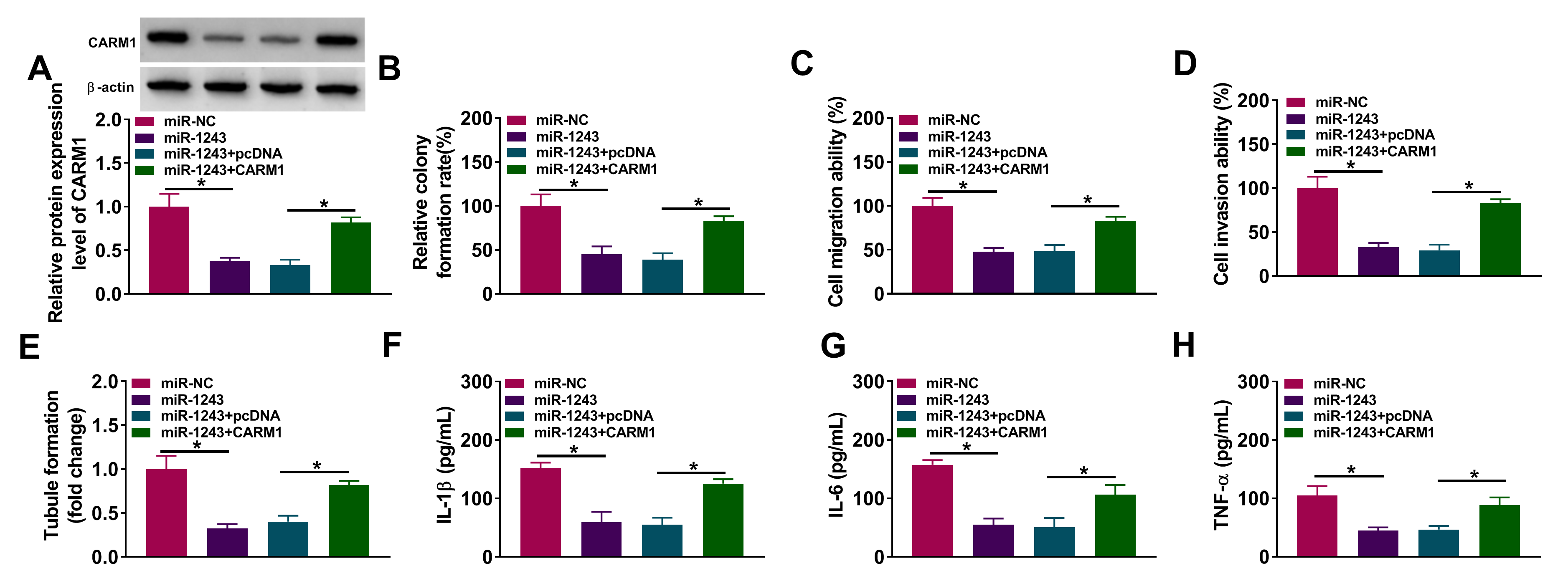

Supplement: Supplementary file 4 — Additional file 4: Figure S4. CARM1 reversed the repressive effect of miR-1243 on high glucose-induced hRMECs function. Following transfection with miR-1243 or/and CARM1, hRMECs were exposed to high glucose for 48 h. Western blot, colony formation, transwell, tube formation and ELISA assays were applied to detect CARM1 protein level (A), cell proliferation (B), migration and invasion (C and D), angiogenesis (E) and pro-inflammatory cytokine release (F–H), respectively. *P < 0.05. [file 13098_2022_787_MOESM4_ESM.tif]
